# Optimal drug combinations and minimal hitting sets

**DOI:** 10.1186/1752-0509-3-81

**Published:** 2009-08-06

**Authors:** Alexei Vazquez

**Affiliations:** 1 The Simons Center for Systems Biology, Institute for Advanced Study, Einstein Drive, Princeton, New Jersey 08540, USA; 2 Department of Radiation Oncology, The Cancer Institute of New Jersey and UMDNJ-Robert Wood Johnson Medical School, 195 Little Albany Street New Brunswick, NJ 08903, USA

## Abstract

**Background:**

Identifying effective drug combinations that significantly improve over single agents is a challenging problem. Pairwise combinations already represent a huge screening effort. Beyond two drug combinations the task seems unfeasible.

**Results:**

In this work we introduce a method to uncover drug combinations with a putative effective response when presented to a heterogeneous population of malignant agents (strains), such as cancer cell lines or viruses. Using data quantifying the effect of single drugs over several individual strains, we search for minimal drug combinations that successfully target all strains. We show that the latter problem can be mapped to a minimal hitting set problem in mathematics. We illustrate this approach using data for the NCI60 panel of tumor derived cell lines, uncovering 14 anticancer drug combinations.

**Conclusion:**

The drug-response graph and the associated minimal hitting set method can be used to uncover effective drug combinations in anticancer drug screens and drug development programs targeting heterogeneous populations of infectious agents such as HIV.

## Background

The main stream in drug discovery has focused on identifying compounds targeting specific malignant agents, such as cancer subtypes or virus strains. In many cases, however, the target of drug therapy is a heterogeneous population of malignant agents, each characterized by a different degree of aggressiveness and response to therapy. Drug resistance is a clear example, whereby an induced or preexisting subpopulation of malignant agents is not responsive to a drug, escaping treatment.

Drug combinations can improve over single therapeuthic agents in two ways. Synergy between two drugs may result in a better response than the two drugs independently. A drug combination may also be more effective when targeting heterogeneous populations of malignant agents. In the latter case, although each single drug may be only effective for a subset of the malignant agents, the drug set as a whole may cover all malignant agents.

Uncovering drug combinations by direct screening is quite challenging due to the large number of potential combinations. A recent high-throughput screen was able to systematically test about 120,000 different two-drugs combinations [1]. Yet, programs like the NCI60 anticancer drug screen count with a stock of above 100,000 potential therapeuthic agents [2], resulting in more than 5 × 10^9 ^two-drugs combinations. The situation becomes even worse when addressing combinations of more than two drugs. More important, assuming that most drug combinations will not improve significantly over single drugs, attempting such high-throughput screens is highly inefficient.

Some interesting techniques are starting to emerge to tackle the potential scarcity of good combinations. The discovery process can be accelerated and the screening costs reduced using stochastic search algorithms and close-loop optimization [3]. Modeling and network approaches can help us to anticipate synergistic effects [4-6]. Yet, there is no general method to identify effective drug combinations from a very large drug stock.

In this work we introduce a systematic framework to uncover effective drug combinations. Our approach is based on the existence of a population of malignant agents (strains), a stock of drugs to target them and certain measure quantifying the response of each strain to each single drug. Starting from this data we construct a strain-drug response graph. Using this graph we show that the problem of finding the minimal number of drugs with a putative effective response over all strains is equivalent to the minimal hitting set problem in mathematics. We illustrate the applicability of this framework using data from the NCI60 anticancer drug screen as a case study. We report 14 drug combinations with a putative effective response over cancer types represented by the NCI60 panel of tumor derived cell lines.

## Results

### Mapping to a minimal hitting set problem

To start addressing the drug combination problem, let us assume we count with a stock of drugs to target different strains that can be found in the patient population. The strains are characterized, in principle, by a different response to the drugs in our stock. Our goal is to find a minimal set of drugs, taken from the available stock, such that each of the strains will respond well to at least one drug in our set.

**Figure 1:**
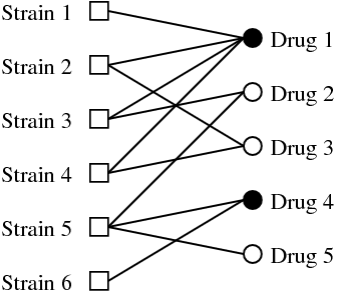
**Strain-drug response graph and the hitting set problem**. A strain-drug response graph with squares representing strains, circles representing drugs, and edges representing a good response of the strain at one end to the drug at the other end. Covered circles represent drugs that are in our cocktail and empty those that are not.

This problem is better understood using the graph representation in Fig. 1. We use one class of vertices (squares) to represent the strains and another class (circles) to represent the drugs. Whenever a strain responds well to a drug we draw an edge between the vertices representing them. In the following we refer to this as the strain-drug response graph. The drug vertices are further divided into covered (filled circles), meaning that they form part of the drug cocktail under consideration, and uncovered (empty circles) otherwise. Now our problem can be rephrased as: determine the minimal number of covered circles (drugs) such that each square (strain) is connected to at least one covered circle, and find such a set (or sets) with a minimal number of drugs. The latter problem is known in the mathematical literature as the *minimal hitting set *problem [7], with strains representing sets and drugs representing set elements.

Let us show how this work in a specific example. The NCI60 is a program developed by the NCI/NIH aiming the discovery of new chemotherapeutical agents to treat cancer [2]. Their drug stock is made of above 100,000 compounds and response data for 40,000 compounds is publicly available. Their population of cancer cell lines (the strains in this context) is made of 60 tumor derived cell lines, representing nine tissues of origin. The cell lines response to the chemical agents is quantified by the IC50, the drug concentration necessary to inhibit the growth of a exposed cell line culture to 50% relative to the untreated control.

To determine what constitutes a good response we use as a reference the IC50 distribution over all pairs (cell line, drug), after performing a z-transformation of the IC50s in a logarithmic scale (Fig. 1a, solid line). This reference distribution peaks at zero and decays very fast beyond two standard deviations. Values to the left denote small sensitivity – bad response – and values to the right denote high sensitivity -good response. In the following we assume as a good response positive values above two standard deviations (Fig. 1a, dashed line). Applying this criteria to each pair of (cell line, drug) we obtain a graph equivalent to that in Fig. 1 for the NCI60 system.

### Finding minimal hitting sets

**Figure 2:**
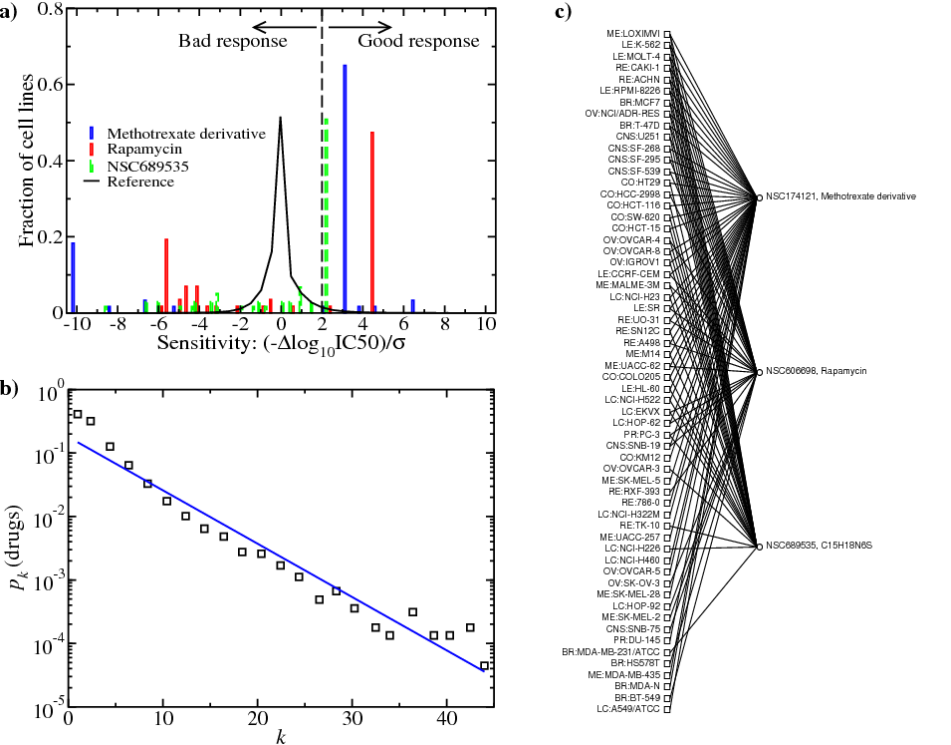
**NCI60 case study**. a) Distribution of the normalized IC50 for three different chemical agents (bars) and the same distribution for all (cell line, drug) pairs (solid line). Δlog_10_IC50 denotes the log_10_IC50 change relative to the drug dependent mean over all cell lines. *s *denotes the standard deviation of Δlog_10_IC50 over all (cell line, drug) pairs. The dashed line marks the threshold at two standard deviations above the mean. b) The fraction *p*_*k *_of drugs connected to *k *strains in the NCI60 strain-drug response graph (symbols). The solid line represents the best fit to an exponential decay. c) Graphical representation of the minimal hitting set number 1 in Table 1.

Having constructed the strain-drug response graph we proceed to identify minimal hitting sets. The hitting set is a computationally hard problem [7]. There is no efficient algorithm to solve it in all possible instances. Yet, using current heuristic algorithms we can estimate the size of the minimal hitting set [8]. In the NCI60 case study, we observe there are some drugs connected to 30 or more cell lines (Fig. 2b).

Covering any of these drugs will automatically reduce to half the size of our computational problem. Thus, we first use a greedy algorithm, first reported in [9], that recursively covers and removes a drug randomly selected among those drugs with the current highest number of connections, until there are no more samples connected to drugs (Methods, highest-degree-first).

Using the greedy algorithm we obtain a hitting set with three drugs. Now three is a sufficiently small number to attempt an exhaustive test of all combinations of one, two and three drugs. In this way we found no hitting sets with one or two drugs, and a total of 14 minimal hitting sets with three drugs (Table 1). The 14 minimal hitting sets were also found using a simulated annealing algorithm (Methods, simulated annealing). The simulated annealing algorithm results in a significant reduction in running time of the NCI60 analysis, from several days to one day in a Desktop computer. It may be used to uncover minimal hitting sets in more computationally demanding problems, where exhaustive test is unfeasible. Fig. 2c shows the graph associated with one of the solutions. It is made of an antimetabolite (NSC174121, methotrexate derivative), a mTOR inhibitor (NSC606698, rapamycin) and a compound of unknown mechanism of action (NSC671526), where NSC stands for cancer chemotherapy National Service Center number. Among the cell lines, 37 out of 60 are covered by more than one drug. Furthermore, the methotrexate derivative is the drug covering more cell lines. This three drug combination looks promising since mTOR inhibitors have been recently shown to work synergistically with methotrexate in the treatment of lymphoblastic leukemia [10]. Further work is required, however, to determine the contribution of the third drug (NSC671526), with currently unknown mechanism of action. Most of the components of the other hitting sets have unknown mechanisms of action as well (Table 2). This is the case for the most recurrent compounds NSC676495 and NSC692745, appearing together in 9 out of the 14 hitting sets. While waiting for further study, our analysis suggests that these are putative effective drug cocktails for anticancer therapy.

**Table 1:** Minimal hitting sets

1	NSC174121	NSC606698	NSC671526
2	NSC174121	NSC606698	NSC689535
3	NSC174121	NSC147340	NSC689535
4	NSC174121	NSC676495	NSC692745
5	NSC21206	NSC676495	NSC692745
6	NSC623794	NSC676495	NSC692745
7	NSC646846	NSC676495	NSC692745
8	NSC656238	NSC676495	NSC692745
9	NSC656240	NSC676495	NSC692745
10	NSC674092	NSC676495	NSC692745
11	NSC682449	NSC676495	NSC692745
12	NSC725983	NSC676495	NSC692745
13	NSC725983	NSC671526	NSC692745
14	NSC606699	NSC689535	NSC691039

All minimal hitting sets for the NCI60 system. NSC stands for cancer chemotherapy National Service Center number. The names and mechanism of action (when available) of these drugs are reported in Table 2.

**Table 2:** Drugs in the minimal hitting sets

NSC	Name	Mechanism of action
21206	6-aminonicotinamide	Antimetabolite
174121	methotrexate derivative	Antimetabolite
606698	Rapamycin prodrug	mTOR inhibitor
606699	Rapamycin prodrug	mTOR inhibitor
147340	Anisomycin hydrochloride	NA
646846	bengamide B	NA
656238	2-Methyl-4,8-dihydrobenzo [1,2-b:5,4-b']dithiophene-4,8-dione	NA
656240	2-Hydroxymethyl-4,8-dihydrobenzo [1,2-b:5,4-b']dithiophene-4,8-dione	NA
671526	Toxin.delta.53L	NA
674092	Quinoline-4-carboxamide, N, N'-[(1,4-piperazinediyl) bis(3,1-propanediyl)]bis(2-phenyl-, dihydrochloride	NA
676495	NA	NA
689535	1-methyl-3-(1-pyrazin-2-ylethylideneamino)-1-(2-pyridin-2-ylethyl) thiourea	NA
692745	1H-Inden-1-one, 2,3-dihydro-2-[(4-hydroxy-3,5-dimethylphenyl) methylene]-5,6-dimethoxy-, (2E)-	NA
623794	1,4-Benzodioxin-2-carboxamide, 6-(4-oxo-4-H-1-benzopyran-2-yl)-N-(3-pyridinylmethyl)-	NA
682449	Benzo [1,2-b:4,5-b']dithiophene-4,8-dione, 2-(1-hydroxyethyl)-	NA
691039	(7S)-7-hydroxy-1,2,3-trimethoxy-10-methylsulfanyl-6,7-dihydro-5H-benzo [a]heptalen-9-one	NA
725983	7-methoxy-5-oxo-8-[3-(9-oxo-9,10-dihydro-4-acridinylcarboxam ido)propoxyl]-(11aS)-1H,2H,3H,5H-bezo [e]pyrrolo [1,2-a][1,4]d iazepine	NA

The list of drugs in the minimal hitting sets reported in Table 1. NSC stands for cancer chemotherapy National Service Center number. NA stands for Not Available.

## Discussion and conclusion

Exhaustive screening of all possible drug combinations is an ineffective strategy to identify good drug combinations. Current screens for single drugs should help to anticipate potentially effective drug combinations, allowing us to narrow down from a see of drug combinations to a short list. The latter can be subject to direct testing, but now with a dramatic decrease of the screening costs.

The strain-drug response graph and the associated minimal hitting set problem provides a systematic framework to tackle this problem. The single agent screen data is represented by a bipartite graph, with a class of vertices representing drugs and another representing malignant agents/strains. Furthermore, the good response of a strain to a drug is represented by a connection between the corresponding vertices in the graph. Using this construction as input, we can search for effective drug combinations, defined as minimal set of drugs such that each strain responds well to at least one drug. The latter problem is mapped to the minimal hitting set problem in mathematics.

The analysis of the NCI60 anticancer drug screen shows how these ideas can be implemented in practice. In this specific example it was possible to identify all minimal hitting sets by exhaustive evaluation of all combinations up to three drug cocktails. An approximate algorithm based on simulated annealing was able to identify all minimal hitting sets as well. The latter algorithm is far more efficient and could be used in problems that are more computationally demanding, with a larger drug stuck or a potentially larger number of drugs in the minimal hitting sets.

**Figure 3:**
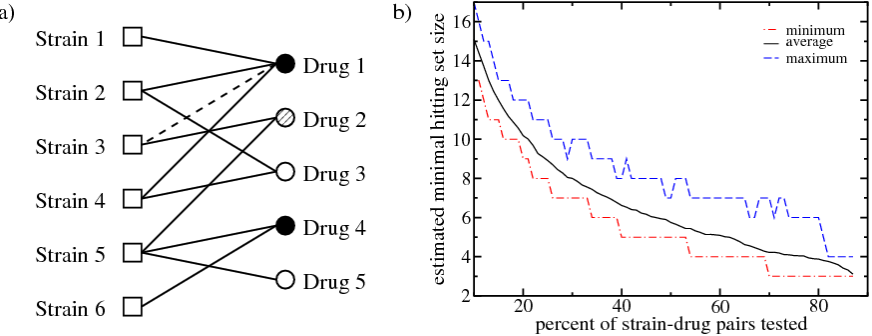
**Minimal hitting sets for incomplete drug-response graphs**. When information about the response of some strains to some drugs is unavailable the strain-drug response graph is incomplete. This could result in an overestimate of the size of the minimal hitting set. a) For example, if the response of strain 3 to drug 1 has not been tested, and the corresponding edge is missing (dashed line), we will be force to cover drug 2. This will increase the minimal hitting set size from 2 to 3 drugs. b) Estimated minimal hitting set size of the NCI60 strain-drug response graph, after assuming that only a certain fraction of the interactions were tested. Note that data for 10% of the strain-drug pairs was already missing from the original dataset and, therefore, we cannot go beyond 90%. The dashed-dotted, solid and dashed line represent the minimum, average and maximum minimal hitting set sizes over 100 incomplete strain-drug response graphs. For each graph the minimal hitting set was estimated using 100 runs of the highest-degree-first algorithm.

The strain-drug response graph and the associated hitting set problem have some caveats. From the technical point of view, we may encounter situations where not all drug-strain pairs have been tested, resulting in an incomplete drug response graph. In this scenario the minimal hitting set size estimated from the incomplete drug-response graph represents and upper bound. This is illustrated in Fig. 3 for the NCI60 analysis. As anticipated above, the estimated minimal hitting set size increases with decreasing the percent of strain-drug pairs tested.

The exhaustive search is not a feasible strategy for very large datasets. Therefore, even when the strain-drug response graph is complete, we would rely on approximate algorithms to obtain an upper bound to the minimal hitting set size. Besides the highest-degree-first and simulated annealing algorithms discussed here, there are other heuristic algorithms [8,11] that in some specific problems may result in better estimates.

From the biological point of view, the identified drug combinations are minimal hitting sets for the NCI60 panel of cell lines. A cell line not included in this panel may not respond well to any of these combinations. Furthermore, using the single drug response data we cannot anticipate potential interactions between the drugs in a given minimal set. Finally, we have not addressed other important issues such as toxicity which may exclude a drug combination for clinical use.

In spite of these caveats, the strain-drug response graph and the associated minimal hitting set problem provide a solid mathematical foundation to the drug combination problem. When information is incomplete and the estimates are approximate, it provides an upper bound to the actual minimal hitting set size. It can be applied to larger panels of cancer cell lines to increase the coverage over the population of cancer cell lines. It narrows down to a short list of drug combinations which can be subject to validation, testing combinatorial effects and toxicity.

In a more general perspective, our formulation can also find applications in drug discovery programs targeting viruses with high mutation rates such as HIV. In this context we would require a collection of virus strains found in the patient population, a stuck of antiviral drugs, and a quantitative measure of how well each virus strain responds to each antiviral drug.

## Methods

### NCI60 data

The IC50 data for the NCI60 panel of tumor derived cell lines was obtained from the Developmental Therapeutics Program of NCI/NIH. It consists of IC50 values for 45,344 compounds against the 60 cancer cell lines.

### Highest-degree-first algorithm

Given a strain-drug response graph, start setting all drugs uncovered. Then recursively transform the drugs state and the drug-response graph as follows: (i) Identify the set of drugs having the largest number of connections in the current drug-response graph. If the latter set is made of one drug select that drug. Otherwise, randomly select one of the drugs in the set. (ii) Set that drug covered, remove the drug, all the samples connected to that drug and the edges connecting the drug and the samples. (iii) Stop if the drug-response graph does not contain any samples connected to at least one drug. Otherwise go to step (i). Note: the application of rule (i) introduces randomness in the algorithm and, as a consequence, different runs may result in different outcomes. Specifically, we may obtain different minimal estimated hitting set sizes and/or different hitting sets with the same size. This fact can be exploited by running the algorithm several times and retaining those solutions having the minimum reported hitting set size.

### Simulating annealing algorithm

Given a strain-drug response graph, introduce the state variable *x*_*i*_, taking the value *x*_*i *_= 1 when element (drug) *i *is covered and 0 otherwise, and the energy or cost function *E *= ∑_*i *_*x*_*i *_counting the number of covered elements. Proceed as follows: (i) Generate a random set cover and set an initial inverse temperature *β *= *β*_0_. The random set cover does not need to be of minimal size. We generate it by covering one element (drug) selected at random from each set (strain) with at least one element. (ii) Perform *T*_eq _equilibration steps. At each step randomly select an element. If it is covered, and uncovering it does not leave uncover any set, then cover it. If it is uncovered, then cover it with probability *e*^-*β*^, where *β *is the equivalent of the inverse temperature in physics. (iii) Increase *β*, *β *→ *β *+ Δ*β*, and return to step (ii). Stop the loop when some convergence criteria is satisfied or *β *= *β*_max_. Note: the generation of the initial state and the application of rule (ii) introduces randomness in the algorithm and, as a consequence, different runs may result in different outcomes. Specifically, we may obtain different estimated minimal hitting set sizes and/or different hitting sets with the same size. This fact can be exploited by running the algorithm several times and retaining those solutions having the minimum reported hitting set size. In the NCI60 study we identified all minimal hitting sets using *β*_0 _= 0, Δ*β *= 0.1, *β*_max _= 20, *T*_eq _= 10 × number of drugs and 1,000 random random covering seeds. A run for each seed took 92 seconds in a 1.86 GHz Desktop computer, 1,000 seeds took 25 and a half hours.

## Authors' contributions

AV conceived and performed the analysis and wrote the manuscript.
